# Programmable all-DNA hydrogels based on rolling circle and multiprimed chain amplification products

**DOI:** 10.1063/5.0169063

**Published:** 2023-10-27

**Authors:** Wildan Hanif, Indresh Yadav, Erol Hasan, Dana Alsulaiman

**Affiliations:** 1 Division of Physical Science and Engineering, King Abdullah University of Science and Technology, Thuwal 23955-6900, Saudi Arabia; 2 Department of Chemical Engineering, Massachusetts Institute of Technology, Cambridge, Massachusetts 02139, USA

## Abstract

Soft, biocompatible, and tunable materials offer biomedical engineers and material scientists programmable matrices for a variety of biomedical applications. In this regard, DNA hydrogels have emerged as highly promising biomaterials that offer programmable self-assembly, superior biocompatibility, and the presence of specific molecular identifiable structures. Many types of DNA hydrogels have been developed, yet the programmability of the DNA building blocks has not been fully exploited, and further efforts must be directed toward understanding how to finely tune their properties in a predictable manner. Herein, we develop physically crosslinked all-DNA hydrogels with tunable morphology and controllable biodegradation, based on rolling circle amplification and multiprimed chain amplification products. Through molecular engineering of the DNA sequences and their nano-/microscale architectures, the precursors self-assemble in a controlled manner to produce soft hydrogels in an efficient, cost-effective, and highly tunable manner. Notably, we develop a novel DNA microladder architecture that serves as a framework for modulating the hydrogel properties, including over an order of magnitude change in pore size and up to 50% change in biodegradation rate. Overall, we demonstrate how the properties of this DNA-based biomaterial can be tuned by modulating the amounts of rigid double-stranded DNA chains compared to flexible single-stranded DNA chains, as well as through the precursor architecture. Ultimately, this work opens new avenues for the development of programmable and biodegradable soft materials in which DNA functions not only as a store of genetic information but also as a versatile polymeric biomaterial and molecularly engineered macroscale scaffold.

## INTRODUCTION

The ability to finely tune the properties of biocompatible hydrogels in a predictable and rational manner opens opportunities for the development of novel application-specific biomaterials for a wide range of advanced biomedical fields from soft robotics and drug delivery to biosensing and tissue engineering.^1,2^ Due to its innate programmability, DNA has been one of the most attractive biological building blocks for the design of programmable architectures based on exploiting highly sequence-specific Watson–Crick base-pairing.^3^ Indeed, the field of DNA nanotechnology has made significant advancements using DNA to form nanorobots^4^ as well as 0D, 1D, and multidimensional structures^5^ assembled via tile assembly^6,7^ and DNA origami,^8^ among other strategies. More recently, DNA has been investigated as a potential building block for the assembly of macroscale polymeric biomaterials, most notably to assemble nanostructured soft hydrogels.^9^ To this end, DNA hydrogels have recently emerged as highly promising three-dimensional self-assembled biomaterials, which can form either physically- or chemically crosslinked networks of nanostructured DNA components.^2^ In addition to the properties of standard hydrogels, including softness, high water content, and tissue-mimicking mechanical properties,^10^ DNA-based hydrogels can also maintain and store genetic information enabling sequence-based programmability^3^ as well as biodegradation,^11^ superior biocompatibility,^12,13^ and controllable self-assembly.^14^ In this way, various designs of DNA hydrogels have been developed and applied to many biomedical applications, including drug delivery,^15^ gene therapy,^16^ tissue engineering,^13,17^ and biosensing.^18^

A major challenge with the development of DNA-based materials is the high costs (i.e., up to thousands of dollars) associated with the synthesis of milligram quantities of DNA required for the construction of macroscale hydrogel networks.^19^ Rolling Circle Amplification (RCA) offers an isothermal enzyme-based approach to synthesize single-stranded DNA (ssDNA) in a low-cost and efficient manner.^20,21^ RCA exploits a polymerase enzyme (commonly phi29) in the presence of a circular DNA template (circDNA) to extend a primer sequence making ultralong ssDNA tandem repeats, complementary to the circDNA template. Yao *et al.* exploited RCA products to form an all-DNA 3D network by hybridizing two RCA products designed to be complementary to each other.^11^ While the latter was developed for stem cell fishing,^11^ other applications of RCA-based hydrogels include biomolecular delivery,^22–24^ bioactive robotics,^25^ and pressure sensing.^14^ Another interesting isothermal approach, called multiprimed chain amplification (MCA), has been introduced to enable subsequent priming of the single-stranded RCA products, thereby forming branched DNA structures.^26^ Lee *et al.* exploited this approach to develop a nest-like structure of DNA hydrogels through physical entanglements of linearly elongated and non-covalently weaved DNA chains.^27^ On the other hand, Merindol *et al.* avoided the additional step of MCA by inserting complementary short ssDNA strands to act as the crosslinker between the strands of RCA products.^14^ Despite promising reports of DNA hydrogels, the immense programmability of the DNA building blocks has not been fully demonstrated. Furthermore, there is a limited understanding of how to rationally tune the properties of DNA hydrogels, and ultimately no established framework to engineer them for particular biomedical applications.

**FIG. 1.**
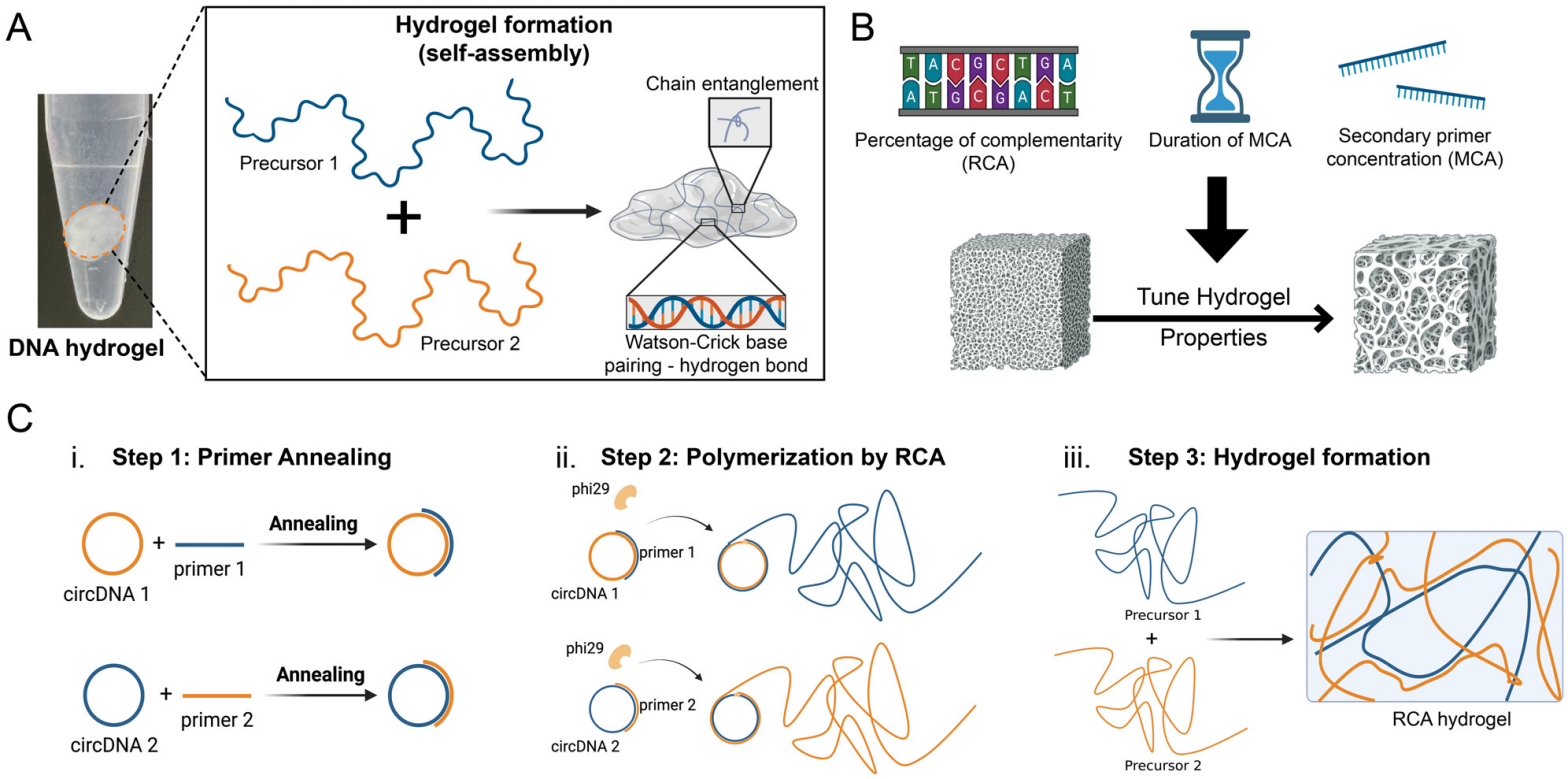
Schematic illustration and photograph of the all-DNA hydrogel biomaterial, self-assembled by incubation of ultralong ssDNA precursors prepared via RCA and/or MCA reactions. (A) Hydrogel formation is driven by two physical interactions: Watson–Crick base-pairing and chain entanglements. (B) Strategies for tuning the hydrogel properties including modulating the percentage complementarity of the precursors, the MCA duration or the secondary primer concentration. (C) Steps outlining the overall DNA hydrogel synthesis methodology, which consists of (i) primer annealing to the circDNA template, (ii) RCA for the generation of the DNA precursors, and (iii) incubation of the two DNA precursors which self-assemble to form the DNA hydrogel.

Herein, we exploit the programmability of DNA nucleotides, based on Watson–Crick base-pairing, to design self-assembling macroscale hydrogels with superior control and tunability over their properties, including microscopic structure, morphology, and biodegradation (Fig. 1). The hydrogels are characterized using scanning electron microscopy (SEM) analysis, and their potential for controlled biodegradation is investigated through digestion assays. Considering their high programmability, tunable morphology, and hydrophilic biodegradable nature, the developed versatile materials offer attractive properties and show promise for use in a range of biomedical applications, especially with the continuously diminishing cost of DNA synthesis.

## RESULTS AND DISCUSSION

Two types of pure DNA hydrogels were synthesized from the enzymatic products of either RCA alone (i.e., RCA hydrogel) or RCA with MCA (i.e., RCA-MCA hydrogels), the products of which self-assemble in a rational manner to produce macroscopic soft hydrogels [Fig. 1(a)]. The novel architectures of the hydrogels were designed through molecularly engineering the ssDNA precursors and their self-assembly process [Fig. 1(b)]. In particular, the properties of the RCA hydrogels were tuned by controlling the nucleotide sequence of the RCA products and, consequently, the percentage of complementarity between the self-assembling hydrogel-forming strands (i.e., hydrogel precursors). For the RCA-MCA hydrogels, we demonstrated a greater degree of control by tuning the MCA duration (i.e., length of DNA precursor branches) and the secondary MCA primer concentration (i.e., number of branches).

### Design of programmable RCA hydrogels

The RCA hydrogels were prepared through a three-step process outlined in Fig. 1(c). First, synthesis of the two ssDNA precursors (shown in orange and blue) involved primer annealing to a circular DNA (circDNA) template (step 1) followed by RCA-based polymerization to form ultralong ssDNA strands with specific sequences (step 2). Next, the precursors were incubated to enable self-assembly and hydrogel formation (step 3). Notably, the entire process has been optimized to only require a total of 9 h from primer annealing to hydrogel formation, which is significantly more efficient than previously reported methods in the literature requiring from 10 h up to 65 h to produce similar millimeter scale DNA hydrogels.^11,14,27^ One elegant way to control the RCA hydrogel properties is by manipulating the degree of complementarity between the two hydrogel precursors, thereby finely tune their interactions at the molecular or sequence level. To this end, we designed four 50-nucleotide (nt) oligonucleotides to act as linear templates for the circDNA used in RCA. Considering circDNA 1 as the original sequence, circDNA 2, circDNA 3, and circDNA 4 were designed to be 100%, 50%, and 25% complementary to the original sequence, respectively (Table S1).

### Synthesis of the ssDNA precursors by RCA

**FIG. 2.**
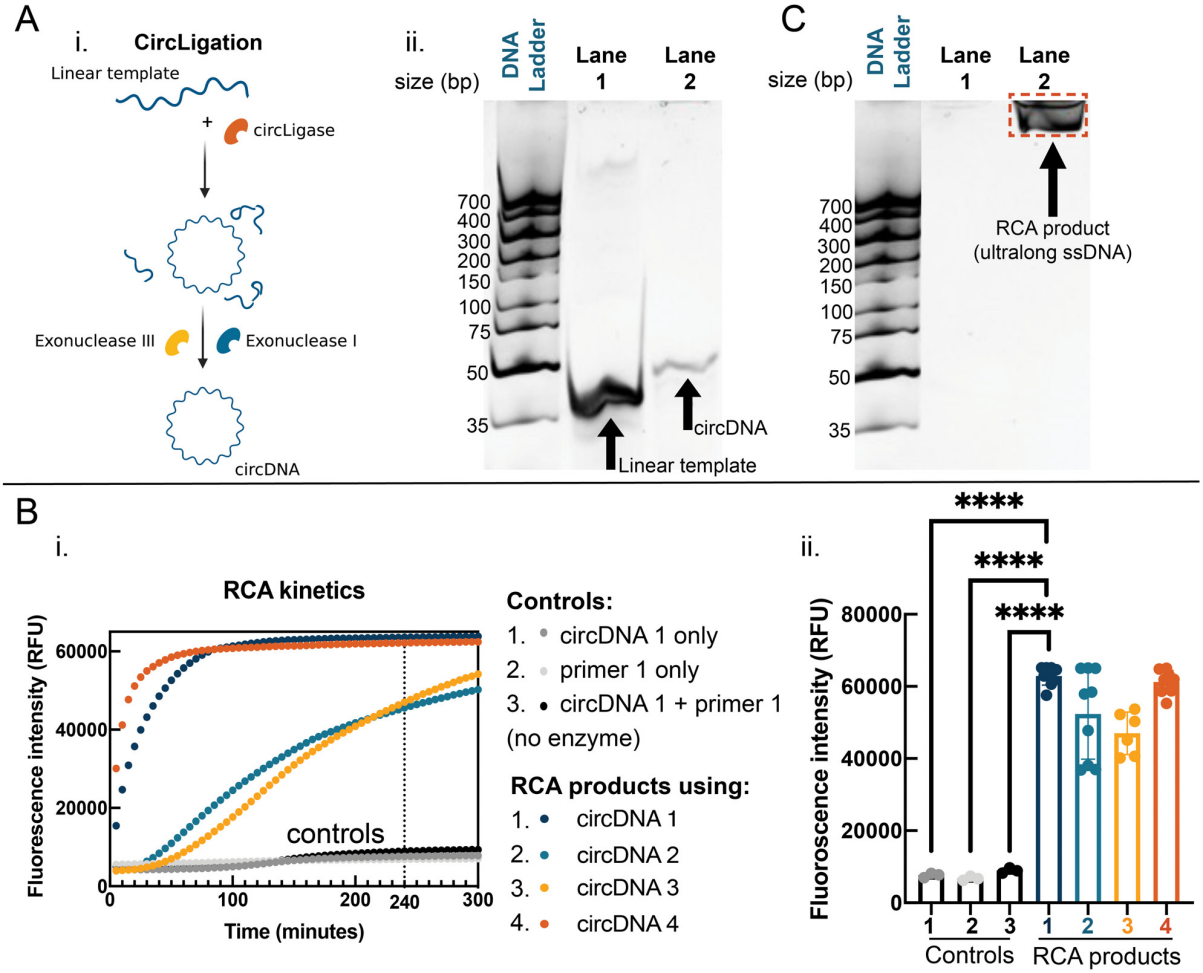
Synthesis and validation of the ultralong ssDNA precursors via RCA-based amplification. (A) (Left) CircLigation step to generate circDNA templates based on enzymatic ligation and purification by exonuclease digestion of circularized fragments. (Right) Gel electrophoresis showing gel-shift in lane 2 indicating successful circularization of the Linear Template (lane 1) (20% PAGE gel, 200 V, 75 min). (B) (i) Fluorescence monitoring of the RCA reaction kinetics, showing that 4 h is sufficient to generate ultralong ssDNA. (ii) Fluorescence intensity of the reaction products after 4 h of RCA confirming the successful generation of ultralong ssDNA precursors (RCA products) compared to the three control reactions (unpaired two-tailed t-test, ^****^indicates p-value < 0.0001). (C) Gel electrophoresis showing the RCA product confined in the well, confirming the success of the RCA reaction in generating ultralong ssDNA (20% PAGE gel, 200 V, 75 min).

Before the first step of primer annealing, the circDNA templates were synthesized based on previously developed protocols of circligation.^28^ Briefly, 50 nt linear templates were purchased commercially and ligated to the circularized form using the enzyme CircLigase II, which catalyzes the intermolecular ligation of the 5′-phosphate and 3′-hydroxyl ends of >15 nt linear ssDNA strands without requiring a template or splint oligonucleotide [Fig. 2(a)].^29^ To ensure a pure circDNA template was developed, the reaction was then purified by treatment with the enzymes, Exonuclease I and Exonuclease III, which digest linear ssDNA and dsDNA strands, respectively. Successful circularization of the linear templates was confirmed by gel electrophoresis on a 20% denaturing polyacrylamide (PAGE) gel. As seen in Fig. 2(a) (ii), compared to the linear template (lane 1), the product after circularization and purification (lane 2) was shifted upwards, indicating lower gel electrophoretic mobility and, thus, successful transformation to the circular configuration.

After successful circDNA synthesis, step 1 of RCA involved annealing a primer to the circDNA template at 45 °C for 1 h. Next, RCA was performed to elongate the primer into ultralong ssDNA strands or hydrogel precursors. To the primer:circDNA complex, the following components were added at the specified concentrations: 1 mM dNTPs, 200 *μ*g/ml BSA, 5 mM dithiothreitol (DTT), and 200 U/ml phi29 DNA polymerase in buffered conditions as detailed in the Methods section. This RCA mix was then incubated at 30 °C on a thermoshaker for 4 h, unless otherwise stated. To develop DNA hydrogels with consistent and controlled properties, we sought to monitor the kinetics of the RCA reaction and characterize the length of the RCA product. Ultimately, the reaction duration was optimized to generate an RCA product with a consistent, ultralong length. To this end, the polymerization reaction was performed in a PCR machine in the presence of SYBR Gold intercalator dye, whose fluorescence signal increases by 1000-fold when bound to ssDNA.^30^ A kinetic study was performed to monitor the growth of the RCA product over time for different circDNA templates, recording fluorescence measurements every 5 min for 5 h. For the polymerization of circDNA 1 and circDNA 4, the reaction reached a plateau signal of roughly 60,000 RFU after 3 h, while a longer time was required for circDNA 2 and circDNA 3 [Fig. 2(b)]. By 4 h, all the curves reached a plateau, indicating the maximum length of the RCA product was established. Consequently, for all following experiments, the RCA reaction was terminated at 4 h, and these RCA products were used for subsequent hydrogel formation and characterization studies.

Successful synthesis of the RCA products was confirmed in two ways. The first approach was by comparing the fluorescence intensity of the products after a 4 h RCA reaction (following incubation with SYBR Gold intercalator) compared to three control reactions containing (i) only circDNA, (ii) only primer, and (iii) circDNA with primer (with no phi29 enzyme). As seen in Fig. 2(b), the fluorescence of the RCA product was significantly greater than the controls indicating the formation of large ssDNA strands (unpaired two-tailed t-test, ^****^indicates p-value < 0.0001). Second, successful polymerization of the RCA products was also confirmed through gel electrophoresis (20% PAGE gel) of the RCA products in the absence (Lane 1) and presence (Lane 2) of phi29 enzyme. As shown in Fig. 2(c), in the presence of the enzyme, the RCA product was formed and remained confined in the well, being too large to be electrophoretically driven through the gel network.

### Self-assembly of the DNA hydrogels—Hydrogel formation

**FIG. 3.**
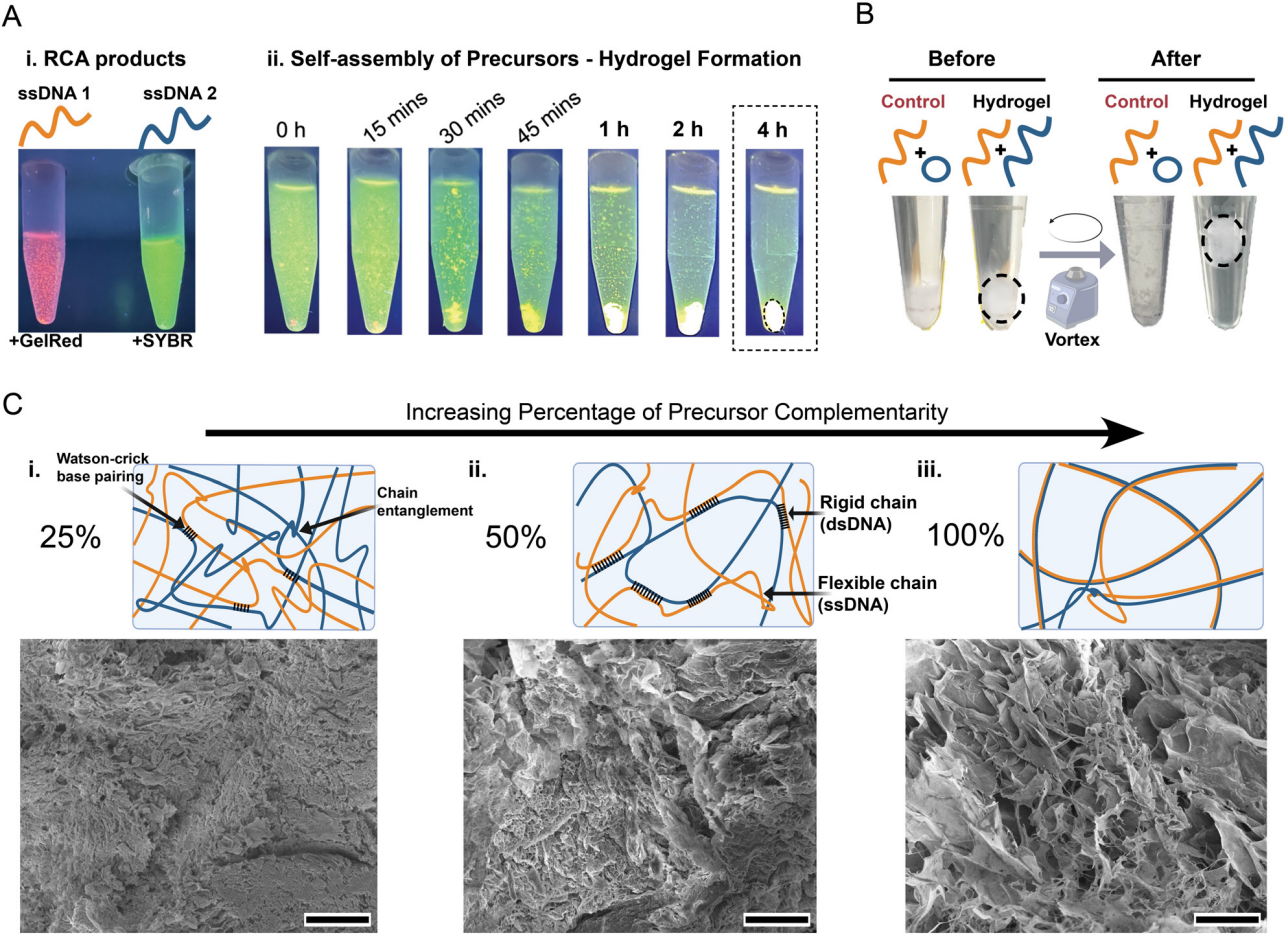
Synthesis and characterization of the RCA hydrogels. (a) Visualization of the gelation process for the DNA hydrogels based on (i) labeling of the two precursors with different nucleic acid dyes followed by (ii) mixing and incubation while monitoring fluorescence emission under UV light irradiation in a dark chamber, showing complete gelation by 4 h. (b) Effect of exposing the control and DNA hydrogel to high shear forces on a vertex mixer, showing the DNA hydrogel maintaining its integrity and structure. (c) SEM micrographs of the three types of RCA hydrogels prepared with precursors of different levels of complementarity (i) 25%, (ii) 50%, and (iii) 100%. Scale bar shows 40 *μ*m.

After enzymatic synthesis of the RCA products or hydrogel precursors (Table S2), the all-DNA hydrogels were self-assembled by mixing two chosen precursors possessing different levels of complementarity between them, as summarized in Table S3. For the RCA hydrogel, the naming convention used hereafter is RCA [percentage complementarity]; for example, RCA 50 refers to the RCA hydrogel made from 50% complementary precursors. For the RCA-MCA hydrogels, the notation used hereafter is MCA [primer concentration in nM] − [MCA duration (h)]; for example, MCA 20 − 1 h refers to the RCA-MCA hydrogel using 20 nM secondary primer and 1 h MCA duration. Hydrogel formation was driven by two processes: (i) hybridization of complementary strands forming hydrogen bonds between the precursors and (ii) molecular chain entanglements of the ultralong precursor strands. Self-assembly of the hydrogels was monitored over time to characterize the gelation process and optimize precursor incubation time. To this end, each precursor was labeled with a dye of different emission wavelengths: either SYBR Gold (λ_ex/em_ of 496/539 nm) or GelRed (λ_ex/em_ of 290/600). UV LED light was then used to excite and monitor the fluorescence emission of the precursors before and after mixing and incubation within a dark chamber. As shown in Fig. 3(a), immediately after mixing the two precursors, we observed the formation of aggregates, which increased in size over time to ultimately generate the macroscale DNA hydrogel network at roughly 4 h from the start of incubation. To confirm hydrogel formation and investigate its mechanical stability, the DNA hydrogel and control condition were subjected to high shear stress in a vortex mixer. The control condition consisted of mixing one precursor with the complementary circDNA and primer (with no phi29 addition). As seen in Fig. 3(b), the DNA hydrogel condition maintained its integrity as a macroscale structure without fragmentation even after excessive shear forces, while the control condition degraded into small fragments.

### Investigating morphology of programmable RCA hydrogels

Using various combinations of ssDNA precursor pairs (Table S3), three types of RCA hydrogels were successfully prepared, each distinct at the nucleotide level: the two precursors were either fully complementary (RCA 100), 50% complementary (RCA 50), or 25% complementary (RCA 25) to each other. Increasing the percentage complementarity would result in DNA hydrogels with increasing ratios of dsDNA compared to ssDNA chains within their network. To investigate the effect of precursor sequence complementarity on the resulting hydrogel structures and morphologies, SEM analysis was performed after lyophilization of the hydrogels. As demonstrated in Fig. 3(c), each of the RCA hydrogels displayed a unique microstructure and morphology. The RCA 25 hydrogel with the largest degree of non-hybridized ssDNA chains exhibited a more dense and uniform structure with the smallest pore size. In contrast, the RCA 100 hydrogel displayed a highly porous and sheet-like structure with a high degree of alignment. Meanwhile, the intermediate condition, RCA 50 hydrogel, exhibited properties of both extremes, showing a dense but porous structure with characteristics similar to the other two hydrogels. The observed trend in the hydrogel structures suggests that a lower degree of complementarity between the precursors results in greater cross-linking and a denser hydrogel. Interestingly, this observation is in agreement with a previous report that studied the mechanical properties of DNA hydrogels, concluding that hydrogels made with a greater degree of ssDNA compared to dsDNA chains had higher stability, a greater cross-linking ratio, and superior mechanical properties.^31^

In essence, the results demonstrate how the rigidity of the precursors can be tuned by changing from single-stranded to double-stranded configuration. This high degree of control has been attributed to the drastic difference in persistence length (L_p_) between these two biopolymers: while dsDNA has an L_p_ of 50 nm (∼150 bp), ssDNA has an L_p_ of 0.75 nm, which is over 60 times smaller.^31–33^ In this way, controlling the percentage complementarity between the precursors enables direct control over the percentage of rigid and flexible chains: the RCA 100 hydrogel would mainly be composed of dsDNA chains, which provide greater rigidity, creating more alignment and directionality in the structure. It is well known that at higher concentrations, dsDNA forms a liquid crystalline structure driven by configurational entropy.^34,35^ Therefore, to maximize the translational degrees of freedom, the fraction of dsDNA chains within the network aligns locally, which is evident in the sheet-like structure of the SEM micrograph for RCA 100. The highly porous aligned morphology of the dsDNA-rich RCA 100 is thus attributed to the rigidity of these chains that hinders conformational chain adjustment during the gelation process, thus minimizing cross-linking density.

As the level of complementarity between precursors is reduced, there is an increase in the percentage of ssDNA chains within the network. These flexible chains can more easily curl or fold over^36^ and undergo conformational adjustment compared to dsDNA chains. For this reason, the flexibility of the ssDNA chains in the RCA 50 and even more so in the RCA 25 network favors a highly entangled and isotropic structure, which ultimately results in a denser network with greater cross-linking density. Overall, the observations herein demonstrate how the properties of RCA-based DNA hydrogels can be tuned in a highly predictable manner to generate hydrogels of varying properties. Notably, the significant differences in the persistence length of ssDNA and dsDNA chains affect the degree of entanglements and local bending rigidity of the network, ultimately influencing the global morphology and mechanical properties of the resulting hydrogel.

### MCA enables more precise control over the DNA hydrogel properties

**FIG. 4.**
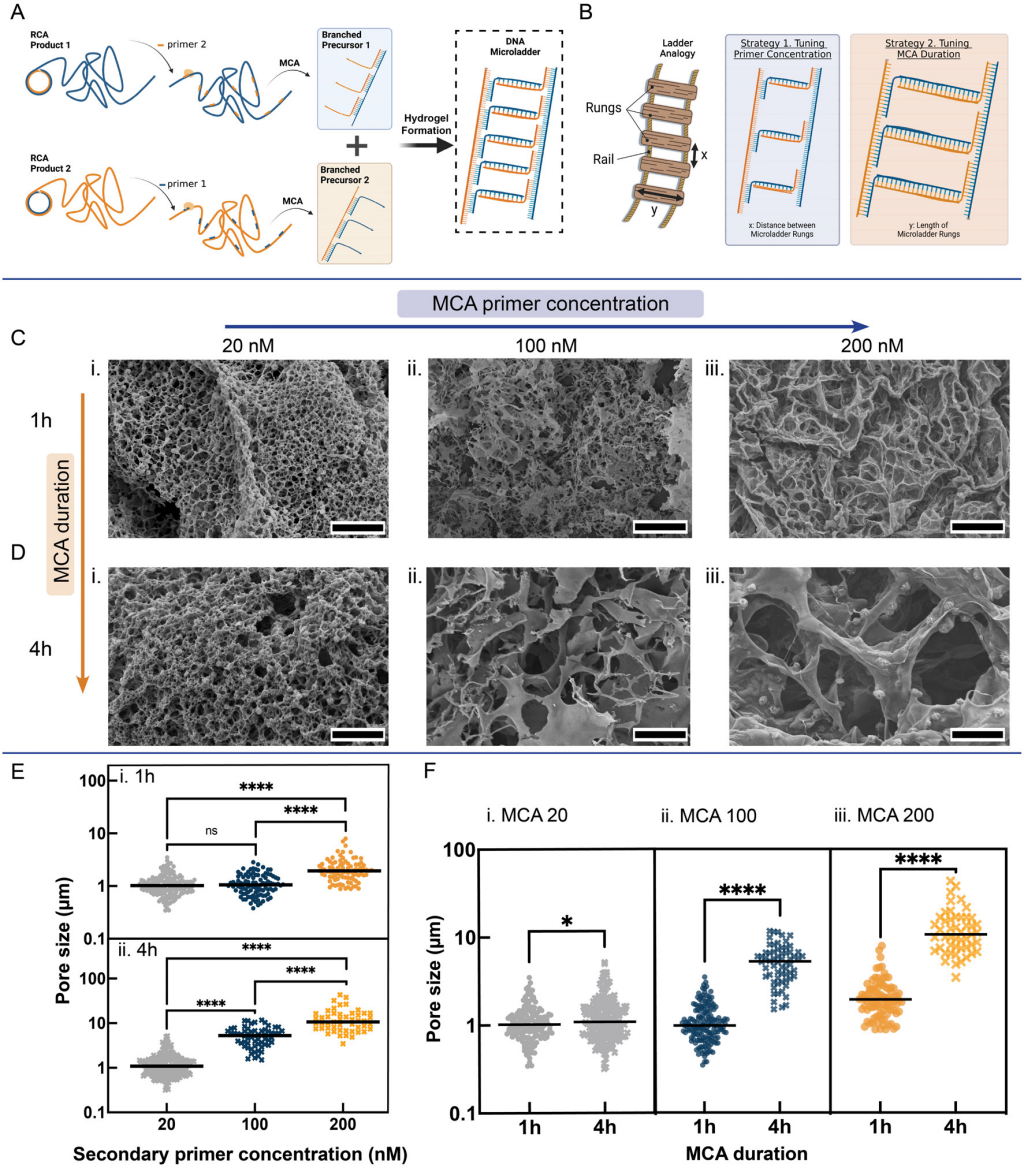
Synthesis and characterization of the RCA-MCA hydrogels. (a) Steps outlining the synthesis of the RCA-MCA hydrogels based on (i) generation of RCA product, (ii) annealing of MCA secondary primer at the desired concentration, (iii) MCA using phi29, and then (iv) incubation of branched precursors enabling self-assembly and hydrogel formation. Hybridization generates DNA microladder-like structures. (b) Ladder analogy to describe the architecture of the hydrogel building blocks, showing the ability to tune the hydrogel by two independent strategies: (1) primer concentration that affects the number of microladder rungs (parameter x) (e.g., effect of increasing x), and (2) MCA duration that affects the length of the individual rungs (parameter y) (e.g., effect of increasing y). SEM micrographs of the RCA-MCA hydrogels prepared at a fixed MCA duration of 1 h (c) or 4 h (d) while varying primer concentration between (i) 20, (ii) 100, and (iii) 200 nM. Scale bar shows 10 *μ*m. (e) Effect of secondary primer concentration on pore size of the RCA-MCA hydrogel, showing a statistically significant increase in pore size with increasing primer concentration (unpaired two-tailed t-test, ^****^indicates p-value < 0.0001). (f) Effect of MCA duration on the pore size of RCA-MCA hydrogel, showing a statistically significant increase in average pore size with increasing MCA duration from 1 to 4 h, across all secondary primer concentrations (unpaired two-tailed t-test, ^****^indicates p-value < 0.0001, ^*^indicates p-value < 0.05). The average pore size (σ) ± standard error of mean for the various hydrogels—based on N pores—are as follows: for MCA 20 − 1 h, σ = 1.1 ± 0.04 *μ*m (N = 164). For MCA 20 – 4 h, σ = 1.3 ± 0.0.6 *μ*m (N = 205). For MCA 100 − 1 h, σ = 1.2 ± 0.05 *μ*m (N = 99). For MCA 100 − 4 h, σ = 5.4 ± 0.3 *μ*m (N = 64). For MCA 200 − 1 h, σ = 2.2 ± 0.1 *μ*m (N = 91). For MCA 200 − 4 h, σ = 13 ± 1 *μ*m (N = 49).

Based on the previous results and to introduce further control over the morphology of the DNA hydrogels, we designed another type of hydrogel, termed RCA-MCA hydrogel, by introducing branches off of the RCA products through the enzymatic process of MCA [Fig. 4(a)]. In this way, we changed the hydrogel precursors from simply ssDNA structures to ssDNA with branches of varying numbers and lengths. These branched precursors were then combined with their complementary counterparts to form the new RCA-MCA hydrogels with a novel ladder-like architecture, which has never been previously reported in the literature and is hereafter referred to as “DNA microladder” [Fig. 4(a)]. Considering a ladder as an analogy for the developed architecture, we sought to investigate the ability to control two distinct parameters: (i) the number of ladder rungs or distance between rungs (parameter x), and (ii) the length of the ladder rungs (parameter y) [Fig. 4(b)]. After synthesis of the ladder “rail” based on a 4h RCA reaction, the number of rungs can be controlled by the concentration of secondary MCA primer. In the presence of phi29 DNA polymerase, the secondary primers are elongated until another MCA primer is encountered. At that point, the strand displacement capacity of the enzyme can cause displacement of the preformed double-strand, leading to branching of the ssDNA chains off the DNA microladder rail [Fig. 4(a)]. Therefore, the length of the DNA microladder rungs can also be controlled by the duration of the MCA reaction. Like the RCA hydrogels, the formation of the RCA-MCA hydrogels was triggered by the incubation and self-assembly of two branched precursor strands. In this case, the branched precursors were designed to be fully complementary to one another (100% complementarity).

We hypothesized that introducing branches onto the main ssDNA microladder rail through MCA would introduce a new architecture and even more sophisticated control over the resulting macroscale hydrogel and its properties. SEM was thus used to investigate the morphology of these hydrogels. Compared to the RCA hydrogels, all the RCA-MCA hydrogels exhibited a porous open-network structure with a more uniform pore size and distribution [Figs. 4(c) and 4(d)]. To study the effect of tuning the number of rungs or inter-rung distance (parameter x), RCA-MCA hydrogels were synthesized with varying concentrations of secondary primer: 20 nM, 100 nM, or 200 nM while maintaining a constant MCA duration of 1 h [Fig. 4(c)]. It is noteworthy that the overall length of the DNA microladder “rail” remains constant as this is controlled by the RCA duration, set to a constant 4h.

As observed in Fig. 4(c), increasing primer concentration resulted in hydrogels with larger pore sizes and a greater alignment of the network structure. This can be attributed to the increase in rigid dsDNA chains of the rungs, which increases the bending rigidity of the DNA microladder. As a result, translational entropy favors the local alignment and liquid crystalline structure of the network, which leads initially to a sheet-like structure [Fig. 4(c-ii)] followed by bundling in 3D [Fig. 4(c-iii)].^37^ To confirm this observation, a similar experiment was performed but with an MCA duration of 4 h (as opposed to 1 h) while varying primer concentration. Notably, the same trend was observed in the SEM micrographs, with an even more drastic and apparent increase in pore size and local alignment of the network with increasing primer concentration [Fig. 4(d)]. On the contrary, decreasing primer concentration [i.e., moving opposite the arrow direction in Figs. 4(c) and 4(d)], results in denser and more crosslinked networks with smaller pore sizes. This is due to the increase in percentage of ssDNA-rich sites [Fig. 4(b)], which can affect the global rigidity of the chain by reducing the local persistence length,^38^ thereby increasing flexibility.

The effect of tuning DNA microladder rung length (parameter y) can also be clearly observed by comparing the SEM micrographs of the hydrogels with the same primer concentration but with varying MCA duration [Fig. 4(c), orange arrow]. In all three cases (20 nM, 100 nM, and 200 nM), increasing MCA duration from 1 to 4 h resulted in a notable increase in pore size, network structure alignment, and rigidity. We attributed this to the greater probability of dsDNA branches in the network in the case of longer MCA duration. The longer branches are more likely to interact, entangle, and hybridize with one another during the gelation process, forming dsDNA microladder rungs with greater rigidity. Ultimately, increasing rung length leads to the net low density of the network, which is analogous to the influence of branching on polymer chains used to create low-density plastics.^39,40^

To confirm the findings through quantitative analysis, the average pore size and pore distribution of the various RCA-MCA hydrogels were extracted from the SEM images using Image J software and plotted, comparing the effect of MCA primer concentration [Fig. 4(e)] and MCA duration [Fig. 4(f)] on hydrogel pore size. The results further confirmed the prior qualitative findings, wherein hydrogel pore size can be significantly increased through two independent strategies: increasing the MCA duration and/or secondary primer concentration. For example, comparing the MCA 20 − 1 h hydrogel to the MCA 200 − 4 h hydrogel, the average pore size could be increased by over one order of magnitude from 1.1 ± 0.04 μm to 13 ± 1 *μ*m, respectively. The level of tunability achieved through the presented approach is exceptional considering hydrogel formation and self-assembly occur in a single pot under aqueous conditions without the requirement for additional chemical crosslinkers, the introduction of leaching agents, or the use of cryogelation approaches. This level of control is rarely observed in conventional polymer-based materials based on (meth)acrylate, (meth)acrylamide, and self-assembling peptides,^41–43^ highlighting the immense potential of this approach in the development of bespoke and versatile biocompatible materials. Due to the limited size of the DNA hydrogel samples, further characterization of the viscoelastic properties of the materials by conventional techniques like bulk rheology was challenging. One potential approach to study these properties for microliter scale DNA hydrogels in future studies is microrheology, which is based on assessing the motion of material-embedded probe particles caused by thermal fluctuations or external forces.^10,44^ These approaches will be investigated and optimized to characterize our hydrogels in future studies.

Overall, the DNA microladder architecture, first presented in this work, introduces much greater control over the hydrogel properties that can be modulated by varying two independent parameters. Tuning these properties in a rational manner, based on the framework developed herein, can be used in the design of highly application-specific hydrogels with defined morphologies and structures.

### Controlled biodegradation of the DNA hydrogels

**FIG. 5.**
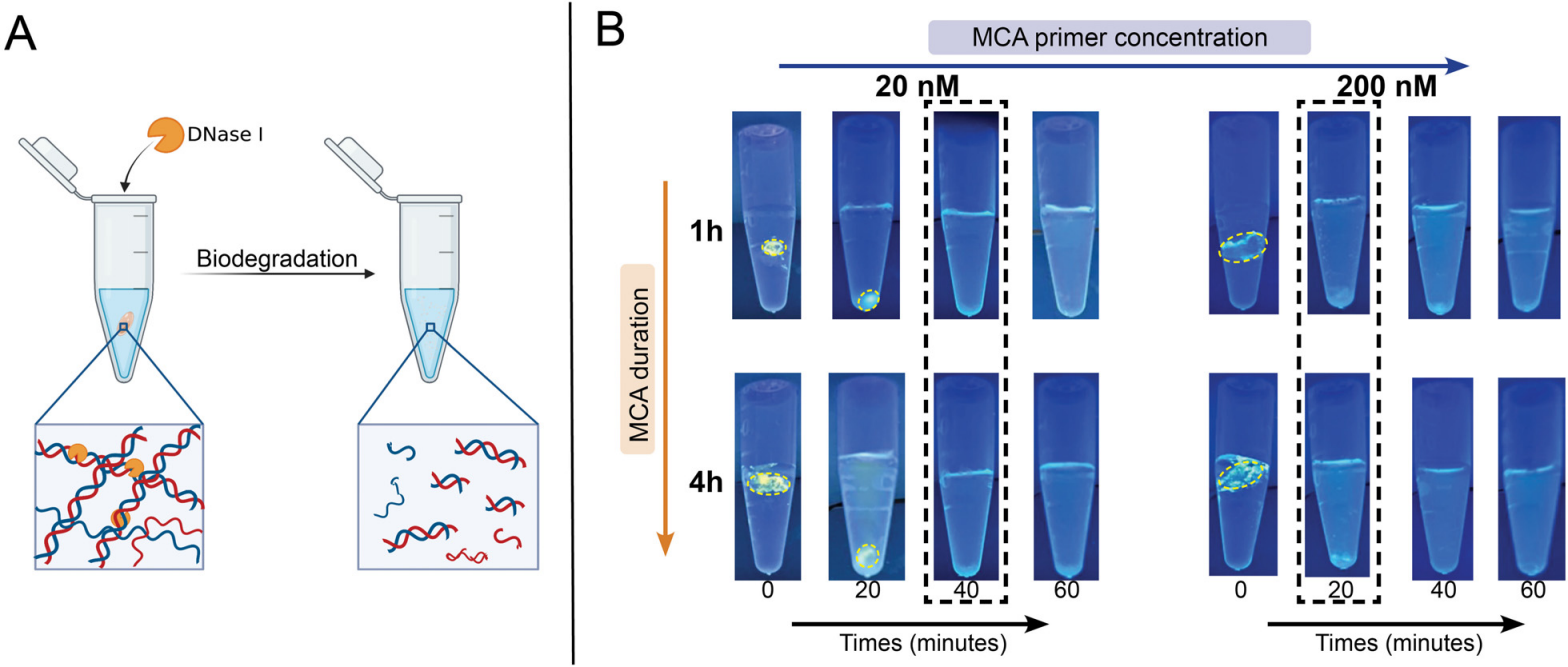
Biodegradation of DNA hydrogel. (a) Schematic showing the biodegradation mechanism of the DNA hydrogels based on DNase I treatment. (b) Monitoring DNA hydrogel biodegradation under UV-LED illumination, comparing the degradation rates as a function of MCA primer concentration and MCA duration. Dashed lines indicate the time at which the hydrogel is fully degraded.

Among the most desirable criteria for biomedical hydrogels are biocompatibility and biodegradability. DNA-based hydrogels, especially exclusively made of DNA, have innate biocompatibility and the ability to be digested under enzymatic control and endogenous conditions.^45^ Deoxyribonuclease (DNase) I is an endonuclease found in the human body, particularly in the digestive system including the pancreatic and parotic systems, as well as in human biological fluids such as blood.^46^ This type of endonuclease is responsible for extracellular waste management by clearing DNA released from apoptotic and necrotic cells.^47^ It works by digesting DNA strands into oligonucleotides with 5′-phospho and 3′-hydroxy ends and, notably, is prone to degrade dsDNA more effectively than ssDNA. We thus sought to study the ability to digest our RCA-MCA hydrogels using DNase I treatment and to investigate if there is a difference in biodegradation rate as a function of their morphology and level of dsDNA within their network [Fig. 5(a)]. To monitor the digestion process *in vitro*, we imaged the SYBR Gold-stained hydrogels under UV-LED illumination in a dark chamber [Fig. 5(b)].

All the hydrogels were completely biodegraded within 40 min from reaction initiation as can be seen in the Eppendorf tubes in Fig. 5(b). However, a notable difference in the overall degradation rate of the hydrogels was observed, wherein the MCA 200 hydrogels possessing a higher concentration of secondary primer, were degraded much quicker than the MCA 20 hydrogels (roughly in 20 min compared to 40 min, respectively). This effect was observed regardless of the MCA duration (1 h or 4 h). This intriguing observation was attributed to two effects that further validate and align with our previous results. First, the MCA 200 hydrogels exhibit a larger pore size that may facilitate the diffusion of the enzyme into the network.^48^ Second, the MCA 200 networks, as previously explained, have a larger proportion of dsDNA chains, which are more susceptible to digestion by DNase I.^46^ This result demonstrates that the developed framework for RCA-MCA hydrogel programming can be exploited to tune the biodegradation rate of the material in a predictable and simple manner. Importantly, this work represents the only demonstration—to our knowledge—of DNA hydrogels with tunable biodegradation properties, which opens new avenues for designing advanced tissue engineering materials and drug delivery systems with controlled release properties and kinetics.

## CONCLUSION

While DNA has been extensively exploited in the field of DNA nanotechnology, developing macroscale materials from DNA building blocks is a more recent and emerging field that has been gaining significant interest. Importantly, through Watson–Crick base-pairing, DNA offers precise control over its monomeric building blocks and unique control over its self-assembly that can subsequently affect its properties at the microscale and macroscale. This work offers a novel well-defined framework for engineering two types of highly tunable and versatile all-DNA hydrogels, called RCA hydrogels and RCA-MCA hydrogels. Interestingly, we demonstrate the ability to finely tune the structure and properties of the DNA hydrogels by modulating three independent parameters: the nucleotide sequence of the precursors, the enzymatic MCA duration, and/or the secondary primer concentration. Importantly, this work is the first demonstration of a DNA microladder architecture that offers an elegant method to control RCA-MCA hydrogels by either modulating the DNA microladder length or the number of microladder rungs. Using the strategies presented, up to one order of magnitude difference in pore size and up to 50% difference in biodegradation rate can be achieved without the requirement for additional crosslinkers, cryogelation processes or leaching agents, commonly exploited in traditional polymeric materials. This superior tunability was enabled by defining the architecture of the hydrogel building blocks and modulating the percentage of ssDNA and dsDNA chains forming the hydrogel networks. This work demonstrates how the significant difference between the persistence length of ssDNA and dsDNA chains can affect the degree of entanglements and local bending rigidity of the networks, resulting in hydrogels with varying properties as studied by SEM analysis and biodegradation assays. Notably, this study represents the only demonstration—to our knowledge—of DNA hydrogels with tunable biodegradation. Owing to their tunability, controllable biodegradation and hydrophilic nature, the RCA and RCA-MCA hydrogels can lay the foundation for the development of advanced and precisely engineered scaffolds for drug delivery and tissue engineering, among other applications. Importantly, the approaches and framework presented can inspire further innovative ways to tailor the properties of DNA hydrogels, thereby enhancing their applicability and meaningful adoption in advanced biomedical applications.

## METHODS

### Materials

All linear DNA templates were purchased from Integrated DNA Technologies (IA, USA). CircLigase II, CircLigase II buffer, Betaine (5M), MnCl_2_, and nuclease-free water were purchased from Biosearch Technologies (Hoddesdon, UK). ATP (10 mM) and dNTPs (10 mM) were purchased from Thermo Scientific (MA, USA). Exonuclease I (10 U), Exonuclease III (100 U), NEB2 buffer (10×), BSA (20 mg/ml), and phi29 DNA polymerase (10 000 units/ml) were purchased from New England Biolabs (MA, USA). DTT (1M), SYBR Gold (10 000×), and ROX reference dye were purchased from Invitrogen (MA, USA). TE buffer (100×) and GelRed Nucleic Acid Stain (10 000× DMSO) were purchased from EMD Millipore (MA, USA). Nuclease-free water was purchased from Omega Bio-tek (GA, USA). Sodium chloride powder and Tween-20 were purchased from Sigma Aldrich (USA).

### Preparation of circDNA templates

Circularization of linear template oligonucleotides into circDNA was conducted by combining 4.17 *μ*M linear template, 1× circLigase buffer, 2.5 mM MnCl_2_, 5 U/*μ*l of circLigase enzyme, 1M Betaine, and 0.167 mM ATP in nuclease-free water to a final volume of 60 *μ*l in an Eppendorf tube. The reaction solution was vortexed briefly and incubated at 60 °C for 1 h on a thermoshaker (Thermo Scientific, MA, USA) with shaking at 1000 rpm. The solution was then incubated at 80 °C for 10 min to deactivate circLigase II and cooled down on ice for 1 min. To digest the uncircularized DNA sequences, 0.08 U of Exonuclease I and 1.67 U of Exonuclease III were added to the same tube, followed by incubation in a thermoshaker at 37 °C for 45 min. Afterward, the temperature was increased to 80 °C for 1 min to deactivate Exonuclease I, then kept at 70 °C for 30 min to deactivate Exonuclease III. For storage purpose, the tube was kept in a −20 °C freezer (for up to 6 months). Before use, the tube was thawed, vortexed briefly, and spun down on a microcentrifuge for 45 s. The desired amount of circDNA template was then pipetted out from the top of the solution.

### Preparation of ultralong ssDNA strands (RCA product)

Two different buffers were initially prepared. TET buffer was prepared by mixing 1× Tris-EDTA (TE) with 0.05% v/v Tween-20. Rinse buffer was prepared by adding 50 mM NaCl to the TET buffer. To anneal the primer to the circDNA template, 0.1 *μ*M primer 1 was combined with 0.1 *μ*M of circDNA 1 in rinse buffer to makeup a solution with a final volume of 50 *μ*l. This solution was then incubated on a thermoshaker at 45 °C and 1350 rpm for 1 h. The RCA mix was prepared by adding the following components into an Eppendorf tube at a final concentration of 1× TET, 1× NEB2, 1 mM dNTPs, 200 *μ*g/ml BSA, 5 mM DTT, and 200 U/ml phi29 DNA polymerase. Next, 200 *μ*l of RCA mix was added to 50 *μ*l of annealing solution and mixed briefly. The solution was then incubated on a thermoshaker at 30 °C and 1350 rpm for 4 h. For the RCA-MCA hydrogels, an additional step of MCA was performed to create branches off the RCA products. To this end, 10, 100, or 200 nM of primer 2 was then added to the reaction tube following RCA to create branched precursor 1 with different numbers of microladder rungs. The MCA reaction was incubated for either 1 h or at 30 °C and 1350 rpm depending on the desired microladder rung length. Finally, the mixture was heated to 65 °C for 10 min to deactivate the phi29 enzyme.

### Kinetics of RCA reaction

The kinetics of the RCA reaction was investigated optically was by conducting RCA reactions in a PCR machine (BioRad, USA). A solution consisting of 0.024 *μ*M circDNA, 0.024 *μ*M primer, 1× TET, and 0.12× NEB2 was prepared in a tube and placed on a thermoshaker at 45 °C for 1 h to enable primer annealing to the circDNA template. An RCA mix was prepared by adding 200 *μ*g/ml BSA, 5 mM DTT, 1 mM dNTPs, 1× SYBR Gold, and 1× ROX dye reference into wells of a PCR plate, with 4U of phi29 added last to initiate the polymerization reaction. The reaction was incubated in the PCR instrument at a constant 30 °C for 5 h, taking fluorescence readings every 5 min. The reaction was conducted in triplicate.

### Gel electrophoresis

Each sample was mixed with 6× loading dye and run on a 20% PAGE/8 M urea denaturing gel at 150 V for 75 min. TriDye Ultra Low Ladder from New England Biolabs (MA, USA) was used as a ladder reference.

### Synthesis of DNA hydrogel

Two hydrogel precursors whose sequences are complementary to each other were mixed in an Eppendorf tube (see Table S3 for list of precursor pairs). The mixture was then incubated on a thermoshaker at 37 °C and 650 rpm for 4 h. A macroscale hydrogel was formed within the tube and stored at 4 °C until use for further experiments.

### Visualization of hydrogel formation

ssDNA 1 and ssDNA 2 were first stained with SYBR Gold and GelRed. Then, they were mixed in the same Eppendorf tube and incubated on a thermoshaker at 37 °C and 650 rpm for 4 h. For visualization purposes, the tube was exposed to UV light at a designated time point (0, 15, 30, 45, 60, 120, 180, and 240 min) and captured for an image.

### SEM analysis

The all-DNA hydrogel samples were frozen at −80 °C overnight before lyophilization in a freeze dryer for 24 h. The freeze-dried samples were then coated with platinum (3 nm). SEM analysis was performed on a Zeiss Merlin instrument (Carls Zeiss, Germany). Quantitative analysis of the pore size was performed using Image J software, and plotting was conducted on GraphPad Prism 9. Statistical tests of unpaired two-tailed t-tests were performed on GraphPad Prism 9.

### Biodegradation studies

All DNA hydrogel samples were stained with 1× SYBR Gold in 200 *μ*l of nuclease-free water at 40 °C and 400 rpm for 40 min. The digestion solution consisted of 1 U of DNase I and 1× DNase I buffer in nuclease-free water at a final volume of 200 *μ*l. The stained sample was then added to the digestion solution and incubated at 37 °C in a thermoshaker to facilitate biodegradation. The biodegradation was observed under a UV lamp with an excitation wavelength of 303 nm, and images were captured at specified time points (0, 20, 40, and 60 min).

## SUPPLEMENTARY MATERIAL

See the supplementary material contains nucleotide sequences for all oligomers used in this manuscript.

## Data Availability

The data that support the findings of this study are available within the article and its supplementary material.
